# Metabolic Bone Diseases Affecting Tooth Eruption: A Narrative Review

**DOI:** 10.3390/children11060748

**Published:** 2024-06-20

**Authors:** Christianna Iris Papadopoulou, Iosif Sifakakis, Symeon Tournis

**Affiliations:** 1 Metabolic Bone Diseases, School of Medicine, National and Kapodistrian University of Athens, 14561 Athens, Greece; irispapad@med.uoa.gr; 2 Department of Orthodontics, School of Dentistry, National and Kapodistrian University of Athens, 11527 Athens, Greece; 3 Laboratory for the Research of Musculoskeletal System “Th. Garofalidis”, School of Medicine, National and Kapodistrian University of Athens, 14561 Athens, Greece; stournis@med.uoa.gr

**Keywords:** tooth eruption, metabolic bone diseases, delayed eruption, tooth impaction, failure of eruption

## Abstract

Tooth eruption is an essential process for the development of the oral and maxillofacial system. Several inherited and acquired diseases might affect this tightly regulated process, resulting in premature, delayed, or even failed tooth eruption. The purpose of this article is to review the literature and the clinical parameters of metabolic bone diseases that affect tooth eruption. It examines the physiological aspects of tooth eruption and the pathophysiological changes induced by metabolic bone diseases, including changes in bone metabolism, density, and structure. The search strategy for this review included an electronic search in PubMed, Google Scholar, Medline, Scopus, and the Cochrane Library using the following keywords: “metabolic bone diseases”, “tooth eruption”, “delayed tooth eruption”, and each reported disease in combination with “tooth eruption disorders”, covering publications up to March 2024 and limited to English-language sources. Understanding the influence of metabolic bone diseases on tooth eruption is crucial for managing both dental and skeletal manifestations associated with these disorders. This review suggests that a multidisciplinary approach to treatment may significantly improve oral outcomes for patients suffering from such conditions. Clinicians should be aware of the specific dental abnormalities that may arise and consider comprehensive evaluations and individualized treatment plans. These findings underscore the need for further research into targeted therapies that address these abnormalities.

## 1. Introduction

Tooth eruption (TE) is a complex process, defined as the emergence of a tooth from its initial position within the alveolar bone to its functional position within the oral cavity. The eruption process continues even after contact with the opposing teeth [1] and is regulated by a variety of genetic, hormonal, and environmental factors. It is a timely, programmed developmental process that requires both bone resorption and formation [2]. However, in individuals with metabolic bone diseases (MBDs), the normal sequence and timing of TE can be disrupted, which often leads to dental abnormalities, such as premature eruption, delayed tooth eruption (DTE) or failure of tooth eruption (FTE) [3].

MBDs are a group of conditions that affect bone metabolism, bone mass, and bone quality. These conditions lead to abnormalities in bone mass, structure, and microarchitecture, which affect bone strength and increase the likelihood of fractures. Consequently, there is a heightened risk of serious complications associated with these diseases [4]. The diagnosis and early treatment of these diseases are vital for maintaining bone health.

These diseases include a variety of syndromes, which have been shown to cause DTE or even FTE, such as cleidocranial dysplasia, Apert syndrome, Gardner syndrome, and amelogenesis imperfecta [5,6,7,8]. Differential diagnosis must exclude primary failure of eruption (PFE), which involves the partial or complete failure of initially non-ankylosed teeth to erupt due to a disrupted eruption mechanism [9]. Moreover, it is essential to exclude other types of eruption failures, such as mechanical failures of eruption (MFE) [10] and isolated ankylosis, which is characterized by infraocclusion, immobility, a metallic sound upon percussion, and radiographic signs including the obliteration of the periodontal ligament space [11].

This review provides a comprehensive and clinically oriented examination of MBDs and their impact on the TE process, beginning with an in-depth examination of the theories and mechanisms underlying TE. Although the impact of MBDs on bone health has been extensively investigated, their effect on TE is not yet fully understood. Understanding the correlation between MBD and TE may provide insight for treating clinical eruption disorders, by enabling more accurate diagnoses and individualized treatments. Additionally, the pathophysiological mechanisms through which MBDs affect TE were analyzed, suggesting potential areas for future research and clinical intervention. Therefore, the aim of this review is to summarize the literature on the effects of MBDs on TE, with the intent of deepening our understanding of how these conditions may alter normal TE, thus providing a comprehensive guide for clinicians to diagnose and manage these disorders.

### 1.1. Pre-Emergent Eruption

TE can generally be divided into pre-emergent and post-emergent eruption phases. The pre-emergent phase involves intraosseous and extraosseous stages. While the crown is still forming, the tooth remains in the same location. Eruptive movements begin with the onset of root formation, causing the tooth to move away from the point at which the root is developing. The eruption path is created by resorption of bone over the root of the permanent tooth simultaneously with TE. An eruptive force is generated, which forces the tooth to move along the eruption pathway, accompanied by bone formation beneath the root [12].

The dental follicle (DF), a loose connective tissue between the alveolar bone and the tooth, regulates TE by controlling osteoclast and osteoblast activity, primarily during the intraosseous phase [13]. It provides the master regulators of bone metabolism, namely,

- colony-stimulating factor-1 (CSF-1);
- receptor activator of nuclear factor kB (RANK) as a receptor and receptor activator of nuclear factor kB-ligand (RANKL) to stimulate osteoclast precursors and induce osteoclastogenesis and osteoclast activity, enabling a major osteoclastogenic spurt to occur;
- osteoprotegerin (OPG), which binds to RANKL and limits its action [2,14].

The production of RANKL and OPG, which are essential for root resorption, is influenced by hormones, such as vitamin D, PTH, and cytokines, which regulate tooth movement toward the alveolar crest and oral cavity [15,16,17].

The extraosseous phase of eruption involves tooth movement from the apex of the alveolar crest through the mucosa to the oral cavity and then to its functional position. During its movement through the oral mucosa, the enamel epithelium merges with the oral epithelium, aiding eruption, while the periodontal ligament (PDL) collagen fibers reorient to support the tooth against occlusal forces. PDL disorders can cause FTE beyond this stage if no physical barrier is present [18,19]. Several TE theories regarding the origin of the eruptive force have been suggested.

### 1.2. Theories of Tooth Eruption

Understanding the molecular mechanisms underlying TE is essential for clinicians to manage related dental complications. Several TE theories involving hydrostatic pressure, root formation, selective bone deposition/resorption, the PDL, and the role of the dental follicle have been proposed [20,21]. The hydrostatic theory, one of the oldest TE theories, suggests that the blood pressure within the vascular tissue between a developing tooth and its surrounding bone generates a mechanical force that drives tooth eruption. Several studies have proven this theory to be inconclusive [22,23,24,25,26]. The theory that root formation results in tooth eruption seems plausible since both processes happen simultaneously; however, studies in dogs and mice have shown that rootless teeth can still erupt and that teeth can sometimes erupt to a greater distance than the length of their root [13,14,18,27,28,29].

Fibroblasts and collagen fibers in the PDL have long been implicated in generating the eruptive force for tooth eruption. Studies have proposed that the proliferation and occlusal migration of these fibroblasts are key factors in this process [30,31,32,33]. Despite the importance of collagen and its remodeling, it cannot be considered the sole mediator of tooth eruption, since teeth without PDL can still erupt. However, the PDL may play a role in the extraosseous phase by lifting the tooth into the occlusal plane [2,12,34]. The bone remodeling theory suggests that bone resorption occurs coronally while bone apposition occurs apically, with the DF being the source of osteoblasts and osteoclasts. Experiments in dogs showed that an eruptive pathway still forms within the bone even when the developing premolar is removed or eruption is prevented, provided the dental follicle remains intact; however, if the dental follicle is removed, no eruptive pathway forms, indicating its crucial role in programmed bone remodeling [21].

Paracrine signaling within the dental follicle during TE involves a series of molecular events set in motion by epithelial–ectomesenchymal interactions among the DF, the reduced enamel epithelium (REE), and the stellate reticulum [35], which release interleukin-1α (IL-1α) to attract monocytes and stimulate osteoclast differentiation [36,37]. This process recruits monocytes to the DF, leading to osteoclastogenesis and bone resorption. The REE and stellate reticulum cells release various signaling molecules, such as CSF-1, monocyte chemotactic protein-1 (MCP-1) and parathyroid hormone-related protein (PTHrP), that attract monocytes and promote osteoclast differentiation, essential for creating an eruption pathway [38,39,40]. Concurrently, the basal DF cells express bone morphogenic proteins (BMPs) 1 and 2, which are greatly enhanced by tumor necrosis factor-a (TNF-α) expression in the dental follicle cells [41]. Further studies are required to fully elucidate the reciprocal signaling between epithelial root sheath cells and the associated DF during the eruptive process. Understanding these mechanisms is crucial for comprehending the subsequent phases of TE.

### 1.3. Post-Emergent Eruption

TE continues even after the tooth comes into contact with its antagonists, slowing and causing tooth wear over time while still maintaining functional occlusion. It compensates for the vertical growth of the jaws, with increased activity during puberty. Therefore, bone metabolism plays a major role in TE [12].

### 1.4. Bone Metabolism and Tooth Eruption Disorders

TE disorders range from tooth impactions caused by physical barriers in the eruption path, such as supernumerary teeth, cysts, or tumors, to submerged teeth that become ankylosed in the alveolar bone and fail to further erupt. Ankylosis refers to the fusion of ostein or dentin with alveolar bone, with a concomitant loss of the periodontal ligament (PDL). Primary failure of eruption (PFE) occurs when non-impacted teeth fail to erupt without the presence of a physical barrier and is related to a malfunction of the eruption process [3,42,43,44].

Disturbances in tooth development are generally categorized into two types of abnormalities. The first type includes structural, morphological, and positional abnormalities, which often require dental treatment, and the second type includes abnormalities in the timing of tooth formation, including disorders such as DTE [3,45]. MBDs may affect TE through conditions such as craniofacial dysostosis, thyroid disorders, and several other recognized genetic syndromes, causing FTE or DTE; however, these conditions are not due to physical barriers [12,36]. DTE is defined as the eruption of a tooth at a time later than that established by standards of different races, ethnicities, and genders. DTE depends on (a) the expected TE time, as indicated by chronological age, and (b) the biological eruption, as indicated by the stage of root formation. Chronological DTE occurs when the eruption time surpasses two standard deviations of the expected eruption time of a specific tooth [3,46,47].

## 2. Materials and Methods

An electronic search was conducted on PubMed, Google Scholar, Medline, Scopus, and the Cochrane Library using the following keywords: “metabolic bone diseases”, “tooth eruption”, “delayed tooth eruption”, and each reported disease in combination with “TE disorders”. All databases were searched as far back as they go. The final update of the search was in March 2024. A total of 386 publications, including clinical studies, systematic reviews, reviews, and case reports, were screened. After removing duplicates, 337 records remained. Following the title and abstract screening, 193 articles were selected for full-text review. This resulted in 129 articles being included in the qualitative synthesis. Each research study was evaluated for potential biases using standardized assessment tools, which considered study design, sample size, and potential conflicts of interest.

Publications were initially screened based on titles and abstracts. Full-text reviews were conducted for studies that appeared relevant, and those relevant to MBDs and TE disorders were included in the final review. Ethical approval was obtained for the publication of clinical and radiographic images of patients treated in the Department of Orthodontics, Dental School, National and Kapodistrian University of Athens, Greece (Approval number: 622/14 February 2024). Informed consent was obtained from the patients for permission to publish their records in the present review.

## 3. Results

### 3.1. Nonsyndromic Disorders

The following nonsyndromic conditions that affect TE were identified: hyperthyroidism, hypothyroidism, hypoparathyroidism, pseudohypoparathyroidism, growth hormone deficiency, and sickle cell anemia. Their incidence and associated TE disorders are depicted in Table 1.

#### 3.1.1. Hyperthyroidism and Hypothyroidism

Thyroid disorders during childhood, which are more common in females, can impact the timing of TE [18]. Previous studies have shown that thyroid hormones play a critical role in the regulation of the metabolic activities of mineralized tissues in the orofacial region, including teeth [54,55]. Hyperthyroidism, marked by increased levels of the thyroid hormones triiodothyronine (T3) and thyroxine (T4), can cause increased metabolic rate, altered hormonal regulation, enhanced osteoclastic activity, and changes in the DF and PDL, which can affect the intraosseous and extraosseous stages of eruption, by accelerating TE [56,57]. Hypothyroidism, characterized by a deficiency of thyroid hormones, can be congenital or acquired. It decreases the metabolic rate and osteoblastic activity and can therefore cause dental abnormalities such as enamel hypoplasia, DTE, micrognathia, and open bite due to incomplete development of the mandible and temporomandibular condyles [26,58].

#### 3.1.2. Hypoparathyroidism and Pseudohypoparathyroidism

Nonsurgical hypoparathyroidism, a rare endocrine disorder, is characterized by low parathyroid hormone (PTH) levels, leading to hypocalcemia and hyperphosphatemia. Depending on the specific cause, hypoparathyroidism is associated with tooth hypocalcemia, tooth hypoplasia, tooth agenesis or impaction, DTE, and short roots [59]. Pseudohypoparathyroidism is characterized by the inability of renal epithelial cells at the proximal renal tubule to respond to PTH, causing excess hormone secretion accompanied by hypocalcemia and hyperphosphatemia [60]. It is caused by mutations in the GNAS gene or upstream of the GNAS complex locus, causing TE problems, such as DTE [52,61].

#### 3.1.3. Growth Hormone Deficiency (GHD)

Growth hormone (GH), which is synthesized and secreted by the anterior pituitary gland, affects growth and metabolism [62]. Excess GH leads to gigantism or acromegaly, while deficiency causes growth retardation or dwarfism [52]. Children with GHD typically experience DTE, which can range from about 1 to 1.3 years. This delay is associated with reduced craniofacial growth, particularly affecting the jaw size and structure, which in turn influences how and when teeth erupt [63,64]. Bone metabolism and remodeling—critical processes for TE—are also affected, which can further delay TE. Pituitary dwarfism features include short stature, DTE, small clinical tooth crowns and roots, and slow mandibular growth [65]. GHD may also cause amelogenesis imperfecta (AI). Treatment involves the administration of synthetic GH (recombinant human GH) [66].

#### 3.1.4. Sickle Cell Anemia

Sickle cell anemia, an inherited hematologic disorder, alters hemoglobin, causing erythrocytes to become sickle cell-like, leading to tissue hypoxia and reduced cell lifespan. This can lead to organ damage and a high risk of infections. Oral impacts include paleness of the oral mucosa, lesions in tongue cells, enamel and dentin hypoplasia, pulp calcification, bone lesions, and DTE. This delay in TE can be attributed to the general slowed growth and development associated with chronic illnesses and potentially to localized disturbances in blood flow affecting jaw growth. Studies have also shown the occurrence of mandibular osteomyelitis, hypesthesia of the mandibular facial nerve, and asymptomatic pulp necrosis [67,68].

### 3.2. Syndromic Disorders

The following syndromic conditions that affect TE were identified: amelogenesis imperfecta, Apert syndrome, Carpenter syndrome, cherubism, cleidocranial dysostosis, mucopolysaccharidosis MPS I-H, MPS II, MPS VI, ectodermal dysplasia, GAPO syndrome, Gardner syndrome, Gorlin–Goltz syndrome, McCune–Albright syndrome, osteoglophonic dysplasia, οsteopetrosis, osteogenesis imperfecta, progeria, sclerosteosis, and hypophosphatemic rickets. Their incidence and associated TE disorders are depicted in Table 2.

#### 3.2.1. Amelogenesis Imperfecta (AI)

**Figure 1:**
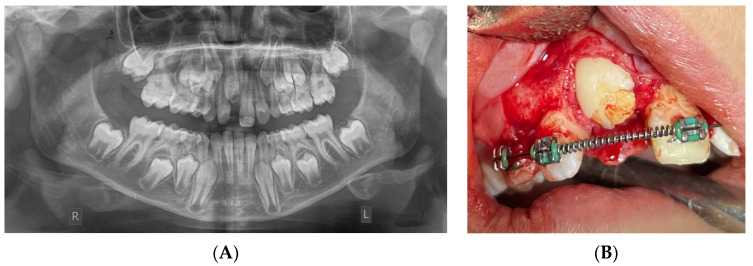
A 9.5-year-old boy presented with impaction of the permanent upper-right central incisor. (**A**) Panoramic X-ray. (**B**) Amelogenesis imperfecta was confirmed during the surgical exposure of the impacted tooth.

AI involves inherited enamel defects with varying phenotypes and genetic patterns. It can be inherited in an autosomal dominant, autosomal recessive, or X-linked recessive manner or can result from spontaneous de novo mutation. There are 4 major phenotypes of AI (hypoplastic, hypomaturation, hypocalcified, and hypomature–hypoplastic enamel with taurodontism), which are further classified into 14 subtypes [69]. In addition to enamel defects, AI may coincide with DTE, FTE (Figure 1), tooth agenesis, short clinical crown heights, malformed teeth, supernumerary teeth, pulp calcifications, taurodontism, root malformations, anterior open bite, and abnormal growth patterns of the jaws, with the hypoplastic type often linked to FTE, presumably due to a defect in the molecular control of eruption [8,87,88,89,90,91].

#### 3.2.2. Apert Syndrome

**Figure 2:**
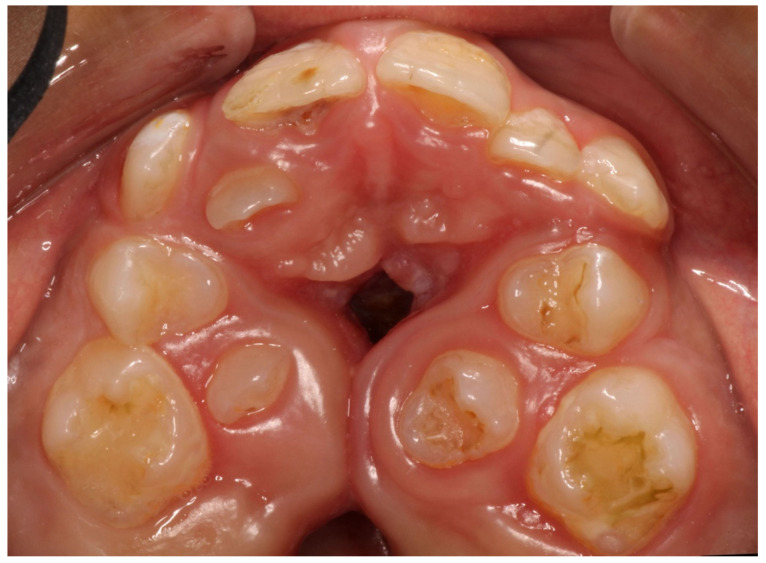
A 12.1-year-old boy with Apert syndrome presented with cleft palate and severe TE disturbances.

Apert syndrome is a rare genetic craniosynostosis disorder (acrocephalosyndactyly type I) caused by FGFR2 gene mutations. It features syndactyly of the hands and feet, premature cranial suture fusion, and an underdeveloped midface, impacting TE [92]. There is typically a 0.96-year delay in TE, ranging from 0.5 to 2.9 years [93,94] (Figure 2). The delayed eruption might be due to the crowding and displacement of teeth within the alveolar bone. Another factor to consider is a primary defect in TE associated with the “mesenchymal disorder” characteristic of Apert syndrome [95].

#### 3.2.3. Carpenter Syndrome

Carpenter syndrome (acrocephalopolysyndactyly Type II), an autosomal recessive craniosynostosis disorder, is characterized by features such as acromegaly, fusion of the fingers and toes, polydactyly, congenital heart disease, intellectual disability, hypogonadism, obesity, and umbilical hernia. It presents with distinct craniofacial features, such as forehead protrusion, wide eyes, a flattened nose, and an underdeveloped midface, which may complicate TE. It is caused by mutations in the RAB23 gene, which regulates the Hedgehog signaling pathway. This pathway plays a significant role in tooth development, affecting the proliferation and differentiation of dental tissues [96]. The syndrome often manifests with a narrow high palate, persistence of primary teeth, hypodontia, and DTE, due to disrupted signaling pathways [97,98].

#### 3.2.4. Cherubism

Cherubism is a rare, autosomal dominant inherited, non-neoplastic disorder causing fibrous connective tissue growth in the jaw bones of children, leading to bilateral, painless, symmetrical jaw enlargement reminiscent of Renaissance cherubs. It mainly affects bone and dental development, causing dental abnormalities, such as DTE, FTE, premature exfoliation of primary teeth, and root resorption [72].

#### 3.2.5. Cleidocranial Dysostosis

**Figure 3:**
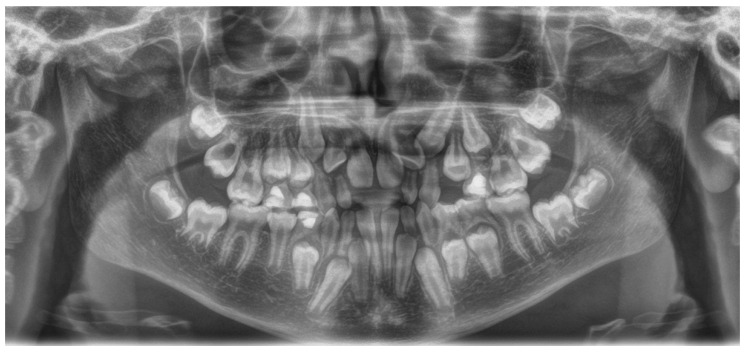
A 11.4-year-old girl with cleidocranial dysostosis presented with multiple impactions of permanent teeth.

Cleidocranial dysostosis is a rare autosomal dominant genetic disorder caused by RUNX2 gene mutations or induced de novo. It is characterized by abnormal clavicles, open sutures and fontanelles, short stature, and other skeletal changes. Oral manifestations include a high, narrow palate, orofacial clefts, DTE impacting teeth during their developmental stages, prolonged primary teeth, and supernumerary teeth (up to 30 teeth) [99,100] (Figure 3).

#### 3.2.6. Mucopolysaccharidosis Type I-H (MPS I-H), II (MPS II), and VI (MPS VI)

Mucopolysaccharidosis types I-H (Hurler), II (Hunter), and VI (Maroteaux–Lamy) are genetic disorders caused by enzyme deficiencies (α-L-iduronidase, iduronate-2-sulfatase, N-acetylgalactosamine-4-sulfatase) leading to glycosaminoglycan build-up, affecting bone metabolism, jaw growth, TE, and potentially life-threatening vital organ damage. This accumulation of glycosaminoglycans in the gingiva and periodontal tissues may result in orofacial and dental abnormalities, including tooth enamel defects, DTE, and the inhibition of tooth development and eruption [101,102,103]. These conditions, especially MPS VI, also contribute to skeletal issues, such as abnormal jaw structure, short stature, cranial dysplasia, increased bone density, bone fragility and deficient bone calcification [102,103,104].

#### 3.2.7. Ectodermal Dysplasia

**Figure 4:**
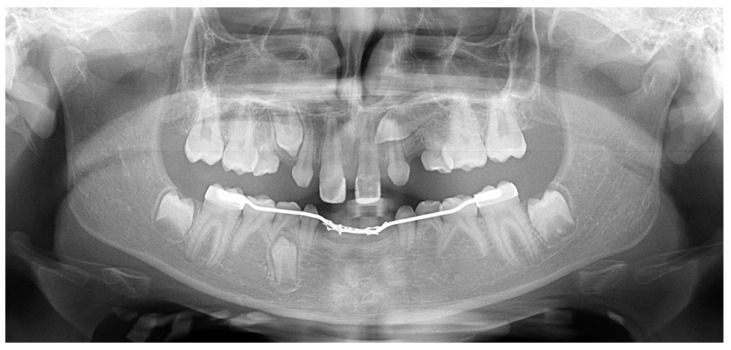
Panoramic X-ray of an 11.8-year-old girl with ectodermal dysplasia presented with severe hypodontia and upper left canine impaction.

Ectodermal dysplasia is a rare metabolic genetic condition that impacts the development of ectodermal organs such as the teeth, nails, hair, and sweat glands. It primarily affects the initial stage of tooth development and often leads to hypodontia, DTE, and abnormal tooth shape, with associated underdeveloped jawbones and a decreased facial height [105,106] (Figure 4).

#### 3.2.8. GAPO Syndrome

GAPO syndrome is an extremely rare autosomal recessive condition characterized by growth retardation, alopecia, pseudoanodontia, and optic atrophy. Affected individuals may exhibit a range of symptoms, including prominent frontal bone, increased intracranial pressure, micrognathia, delayed bone growth, and FTE. The tooth buds seem to develop normally within the alveolar bone, but the teeth fail to erupt into the oral cavity, as the early developmental stages are impacted [107].

#### 3.2.9. Gardner Syndrome

Gardner syndrome, an autosomal dominant familial adenomatous polyposis (FAP) subtype caused by APC (adenomatous polyposis coli) gene mutations, leads to numerous adenomas in the colon and potential colorectal cancer. It has a significant impact on tooth development and eruption due to the abnormal growth of both hard and soft tissues in the oral cavity. Among patients, 30–75% have dental anomalies like tooth agenesis and DTE [7,108].

#### 3.2.10. Gorlin–Goltz Syndrome

Gorlin–Goltz syndrome is a rare autosomal dominant condition with PTCH1 (protein patched homolog 1) gene mutations. It has been known to cause tumor susceptibility (basal cell carcinomas), odontogenic jaw keratinocysts that impact TE, DTE, and bone metabolism disorders influencing posture and bone health [109,110].

#### 3.2.11. McCune–Albright Syndrome (MAS)

McCune–Albright syndrome is caused by de novo GNAS gene mutations at the postzygotic stage. It disrupts skeletal stem cell differentiation due to excess cAMP, leading to skeletal deformities, café-au-lait spots, and endocrine gland hyperfunction. MAS is commonly presented with fibrous dysplasia of the craniofacial bones and supernumerary teeth, leading to impacted TE, DTE, and abnormal bone development [111,112,113].

#### 3.2.12. Osteoglophonic Dysplasia

Osteoglophonic dysplasia, an extremely rare autosomal dominant disorder linked to FGFR1 (fibroblast growth factor receptor 1) mutations. It affects skeletal development, leading to short stature, craniosynostosis, hypertelorism, and brachydactyly. It also affects tooth development and eruption, due to the abnormal growth of both hard and soft tissues, leading to FTE, DTE, and oligodontia [114,115].

#### 3.2.13. Osteopetrosis

Osteopetrosis is a rare skeletal disorder marked by increased bone density caused by osteoclast dysfunction, with a severity ranging from asymptomatic to fatal. Approximately 70% of cases are linked to mutations in 10 genes. It often leads to osteosclerosis, short stature, fractures, cranial base nerve damage, and various dental abnormalities, such as tooth agenesis, impaction, FTE, DTE, enamel hypoplasia, or TMJ dysfunction [116,117].

#### 3.2.14. Osteogenesis Imperfecta (OI)

Osteogenesis imperfecta, or “brittle bone disease”, is predominantly linked to COL1A1 and COL1A2 gene mutations affecting type 1 collagen, which is crucial for bone structure. It is a rare autosomal dominant disease [118]. OI type I is caused by a reduced quantity of collagen. OI types II, III, and IV, classified by the Sillence classification, are caused by qualitative defects in collagen [119]. Approximately 15–20% of cases are attributed to newly identified genes with autosomal recessive inheritance. It mainly affects bones but can also impact other collagen-rich tissues, leading to increased bone fragility, growth disorders, scoliosis, blue sclera, hearing loss, dentinogenesis imperfecta, ectopic TE, hypodontia, oligodontia, tooth impaction, and agenesis [120]. Studies have also shown that OI patients treated with bisphosphonates, such as zoledronate, present DTE, mainly due to the defective collagen matrix [121].

#### 3.2.15. Hutchinson–Gilford Syndrome (Progeria)

Progeria is an extremely rare autosomal dominant condition caused mainly by a specific LMNA gene mutation. This leads to abnormalities in cellular division and collagen remodeling. Early signs include growth retardation, brachysomia and low weight, with disproportionate craniofacial growth, such as micrognathia and retrognathia (with mandibular retrognathia being more prominent). This causes delayed development and eruption of teeth. Patients usually succumb during early adolescence due to cardiovascular disease [122,123,124].

#### 3.2.16. Sclerosteosis

This rare autosomal recessive disorder is caused by mutations in the SOST gene, which encodes sclerostin, a glycoprotein that inhibits bone formation. Sclerosteosis leads to excessive bone growth, gigantism, and syndactyly of the second and third fingers. The bones have increased density and are wider, especially the cranial bones, leading to larger jaws with DTE because of the increased bone density, and orofacial abnormalities such as protruding eyes and forehead and midface hypoplasia [125,126].

#### 3.2.17. Hypophosphatemic Rickets

Hypophosphatemic rickets, a metabolic bone disorder mainly caused by a mutation in the PHEX (phosphate-regulating endopeptidase X-linked) gene, leads to skeletal deformities, growth retardation, and dental problems, affecting all ages and genders. It is characterized by low phosphorus and 1,25(OH)_2_-vitamin D levels, high alkaline phosphatase, and normal or low levels of serum calcium. The signs of this disorder include short stature, musculoskeletal disorders, arthritis, fractures, and various dental abnormalities, such as DTE, enamel hypoplasia, taurodontism, and dental abscesses [127,128].

## 4. Discussion

The present narrative review is a comprehensive overview of the relevant literature on TE disorders associated with MBDs. In order to understand the pathophysiology of these disorders, the clinician must be informed of the various TE stages and how they are regulated. During the pre-eruptive phase, while the tooth is still developing in the alveolar bone, IL-1a and TGF-β1 are the key molecules involved. They are secreted by the stellate reticulum and have a role in signaling the onset of tooth eruption by affecting the DF. At the same time, PTHrP, expressed in the stellate reticulum and later in the DF, is essential for the initiation of TE, and its absence can lead to FTE. During the intraosseous phase, the key molecules include CSF-1—which recruits osteoclast precursors and down-regulates OPG, allowing osteoclastogenesis to proceed—as well as RANKL and OPG. During the extraosseous phase, the main molecules are BMP-2, which promotes alveolar bone growth, as well as TGF-β1 and TNF-α, which play a big role in bone remodeling and up-regulating the RANKL gene expression, further contributing to bone resorption. MBDs that alter the expression of these molecules can alter the TE process, which may result in disorders such as DTE, FTE, and acceleration of TE [2,18,27].

The clinical management of TE disorders in patients with MBDs demands a collaborative, multidisciplinary approach focused not only on achieving optimal tooth alignment and occlusion but on addressing the challenges that each disease poses to bone metabolism. Therefore, a critical aspect of managing TE disorders involves diagnosing and addressing the systemic disease causing the condition, which can significantly influence treatment decisions and outcomes [3].

The main considerations involved an initial assessment, including a comprehensive medical history, diagnostic imaging (orthopantomography) and consultation with the patient’s physician. The treatment strategy must consider whether to retain or extract affected teeth. In cases where tooth development seems normal on radiographic imaging and root development is less than 2/3, observation of the tooth is essential. If the root development is greater than 2/3 and there is no physical obstruction causing the ectopic tooth position, then the treatment options include (a) observation (may self-correct), (b) exposure and orthodontic traction, and (c) extraction and replacement of the tooth (implant, fixed/removable prosthesis, autotransplantation of affected tooth) [3].

Additionally, the existence of physical barriers such as ankylosed or supernumerary teeth and cysts should be evaluated. The treatment strategy may range from removal of the obstruction and observation of the tooth to removal of the obstruction followed by exposure of the affected tooth, exposure plus orthodontic traction, removal of the affected tooth, and replacement of the tooth or orthodontic space closure [43]. Extraction of the neighboring tooth to create space may be an effective treatment option too. Methods of space gaining, such as slow or rapid palatal expansion and interceptive treatment aiming to preserve the E-space (leeway space) should be considered in these cases. Creating and maintaining sufficient space is crucial for successful treatment outcomes [3].

The treatment of hyperthyroidism and hypothyroidism involves distinct approaches adjusted to address the underlying dysfunction. Hyperthyroidism may be managed with antithyroid agents such as propylthiouracil, carbimazole, and methimazole, which inhibit hormone synthesis. Other treatments include iodide, radioactive iodine, or thyroidectomy [57]. Patients with hypothyroidism are typically treated with levothyroxine (T4), which has been shown to induce permanent tooth eruption [58]. In well-controlled patients, standard dental procedures can be followed as with healthy patients. However, it is recommended that situations of high stress and infectious outbreaks are avoided [59].

For patients whose thyroid conditions are not well-regulated, precautionary measures are crucial. Major surgical procedures are generally avoided, because acute oral infections or severe stress can trigger a thyrotoxic crisis in patients with overt hyperthyroidism or exacerbate hypothyroidism in those with inadequate control [58]. Hypoparathyroidism is treated with vitamin D or its analogs, calcium salts, and medications that enhance renal calcium reabsorption to maintain normal serum calcium levels [58].

Patients with acrocephalosyndactyly syndromes, such as Apert and Carpenter syndromes, require an individualized treatment plan tailored to their specific needs. Münster University Hospital has published a comprehensive treatment plan for Apert syndrome, divided into five stages according to age. The plan begins at 3–6 months with an excisional–osteoclastic method. From 7–10 years, it includes complex surgical procedures such as LeFort III osteotomy and frontofacial advancement. At 9 years, the plan suggests treatment involving extractions, followed by orthodontic treatment at 10 years to prepare for orthognathic surgery. Finally, orthognathic surgery is recommended for patients over 16 years of age [93].

For mucopolysaccharidoses, including Hurler, Hunter, and Maroteaux–Lamy syndromes, the treatments of choice include enzyme replacement therapy, hematopoietic stem cell transplant (HPSCT), and conservative management of symptoms. Tooth development disturbances may occur due to the secondary effects of chemotherapy and radiotherapy, such as tooth agenesis and tooth hypoplasia [102].

In patients with ectodermal dysplasia, diagnosis is most effectively made during childhood through medical history and clinical examination. The primary goals of dental treatment for these patients are the restoration of missing teeth and bone, as well as the restoration of normal vertical dimension [106].

Patients with hypophosphatemic rickets show a positive response to treatment with phosphate salts and 1,25(OH)_2_D (calcitriol). Some effects of 1,25(OH)_2_D on dentin may occur indirectly by enhancing the intestinal absorption of calcium and phosphorus. Additionally, 1,25(OH)_2_D may directly influence mesenchymal dental stem cells, as both dentinoblasts and ameloblasts express the vitamin D receptor. Dental management for individuals with hypophosphatemic rickets should include topical fluoride applications, preventive resin restoration (PRR), oral hygiene instructions, and regular check-ups. These measures help to ensure optimal oral health and minimize the potential dental complications associated with the condition [127].

The present study is an updated review, raising awareness of the association between MBD and TE and is thus helpful in daily dental practice. It assessed all English-language studies published up until March 2024. Despite its clinical significance, this study has some limitations. One of the primary limitations is that it was conducted as a narrative review rather than a systematic one. One potential source of bias is the exclusion of articles not written in English, which may have limited the scope of data and perspectives included. Additionally, the variability in clinical presentations and progression of MBDs can make it challenging to develop universally applicable treatment protocols.

Future research methodologies should aim to include larger sample sizes, in order to provide powerful evidence on the relationship between MBDs and TE. More robust methods and modern approaches such as morphometrics may provide further insights into the anatomical and developmental complexities of these disorders. Additionally, integrating advanced imaging techniques and genetic profiling could significantly enhance the understanding and management of TE disorders associated with MBDs.

## 5. Conclusions

Nonsyndromic and syndromic MBDs may affect TE in a variety of ways. Dental care practitioners should be aware of the association between MBDs and TE. Early diagnosis and early attempts to address the systemic disease causing the disorder is a critical aspect in managing TE disorders and influences treatment decisions and outcomes significantly. Collaboration among dental specialists is essential to provide comprehensive care and optimize treatment success for patients with underlying metabolic conditions.

## Figures and Tables

**Table 1:** Nonsyndromic disorders; incidence and associated TE disorders.

Nonsyndromic Disorders	Incidence (Births)	Tooth Eruption Disorder
Hyperthyroidism	1:50–200 [48]	Accelerated TE
Hypothyroidism	1:4000–10,000 [49]	DTE
Hypoparathyroidism	2.3–3:100,000 [50]	DTE
Pseudohypoparathyroidism	0.3–1.1:100,000 [51]	DTE
Growth Hormone Deficiency	1:4000–10,000 [52]	DTE
Sickle Cell Anemia	515,000 (425,000–614,000) births in 2021 [53]	DTE

Abbreviations: TE, tooth eruption; DTE, delayed tooth eruption.

**Table 2:** Syndromic disorders; incidence and associated TE disorders.

Syndromic Disorders	Incidence (Births)	Tooth Eruption Disorder
Amelogenesis Imperfecta	1:700–14,000 [69]	FTE, DTE
Apert Syndrome	1:65,000–200,000 [70]	DTE
Carpenter Syndrome	1:1,000,000 [71]	DTE
Cherubism	~300 cases [72]	FTE, DTE
Cleidocranial Dysostosis	1:1,000,000 [73]	DTE
MPS I-H	1:100,000 [74]	DTE
MPS II	1:100,000 [75]	DTE
MPS VI	0.36–1.3:100,000 [76]	DTE
Ectodermal Dysplasia	7:10,000 [77]	DTE
GAPO Syndrome	~60 cases [78]	FTE
Gardner Syndrome	1:7000–30,000 [79]	DTE
Gorlin–Goltz Syndrome	1:40,000–60,000 [80]	DTE
McCune–Albright Syndrome	1:100,000–1,000,000 [81]	DTE
Osteoglophonic Dysplasia	Unknown	FTE, DTE
Osteopetrosis	1:20,000–250,000 [82]	FTE, DTE
Osteogenesis Imperfecta	0.2–0.7:10,000 [83]	DTE
Progeria	1:20,000,000 [84]	DTE
Sclerosteosis	1:60,000 (Afrikaner) [85]	DTE
Hypophosphatemic Rickets	1:20,000–200,000 [86]	DTE

Abbreviations: FTE, failure of tooth eruption; DTE, delayed tooth eruption; MPS, mucopolysaccharidosis.

## Data Availability

The data presented in this study are available on request from the corresponding author. The data are not publicly available due to restrictions, e.g., privacy or ethical.

## Abbreviations

AI	Amelogenesis Imperfecta
APC	Adenomatous Polyposis Coli
BMPs	Bone Morphogenic Proteins
cAMP	3′-5′ Cyclic Adenosine Monophosphate
CNS	Central Nervous System
CSF-1	Colony-Stimulating Factor-1
DF	Dental Follicle
DTE	Delayed TE
FGF	Fibroblast Growth Factor
FGFR	Fibroblast Growth Factor Receptor
FTE	Failure Of TE
GH	Growth Hormone
GHD	Growth Hormone Deficiency
HPSCT	Hematopoietic Stem Cell Transplant
IL-1α	Interleukin-1α
MAS	McCune –Albright Syndrome
MBD	Metabolic Bone Diseases
MFE	Mechanical Failure of Eruption
MCP-1	Monocyte Chemotactic Protein-1
NGF	Nerve Growth Factor
OI	Osteogenesis Imperfecta
OPG	Osteoprotegerin
PDL	Periodontal Ligament
PFE	Primary Failure of Eruption
PHEX	Phosphate-Regulating Endopeptidase X-Linked
PRR	Preventive Resin Restoration
PTH	Parathyroid Hormone
PTHrp	Parathyroid Hormone-Related Protein
PTCH1	Protein Patched Homolog 1
RANK	Receptor Activator of Nuclear Factor Kb
RANKL	Receptor Activator of Nuclear Factor Kb-Ligand
REE	Reduced Enamel Epithelium
TE	Tooth Eruption
TGF-β	Transforming Growth Factor-Beta
TNF-α	Tumor Necrosis Factor-a
